# Tagging synthetic polymers with coumarin group for study nucleic acid interaction with gene delivery agents

**DOI:** 10.1016/j.mex.2019.01.008

**Published:** 2019-01-24

**Authors:** Elena N. Danilovtseva, Viktor A. Pal'shin, Uma M. Krishnan, Vadim V. Annenkov, Stanislav N. Zelinskiy

**Affiliations:** a Limnological Institute of the Siberian Branch of the Russian Academy of Sciences, 3, Ulan-Batorskaya St., P.O. Box 278, Irkutsk, 664033, Russia; b Centre for Nanotechnology & Advanced Biomaterials (CeNTAB), School of Chemical and Biotechnology, SASTRA University, Thanjavur, 613401, Tamil Nadu, India

**Keywords:** Labeling of polymeric amines with coumarin tag, Poly(vinyl amine), Polyethylenimine, Diethylaminocoumarin-carboxilic acid, Gel electrophoresis

## Abstract

Polymeric amines and complex amine containing system are actively studied and applied as gene delivery agents in gene therapy and genetic engineering. Optimizing polymer – nucleic acid ratio is the key stage in elaboration of procedures in this area. Application of fluorescent tagged oligonucleotides is widespread approach which allows to visualize nucleic acid in gel electrophoresis experiments and to find conditions of the full binding of the nucleic acid. We suggest to use succinimidyl ester of 7-(diethylamino)coumarin-3-carboxylic acid as an agent for fluorescent labeling of polymeric amines and to use the tagged polymers in optimizing polymer – nucleic acid ratio. This approach allows to see unbound polymer and to study various nucleic acids in interaction with the same polymer.

• Labeling of gene delivery agents with fluorescence groups increases efficiency of optimization of gene delivery compositions.
• Polymeric amines tagged with succinimidyl ester of 7-(diethylamino)coumarin-3-carboxylic acid are suitable for study polymer – nucleic acid interaction with gel electrophoresis.

## Graphical abstract

**Figure:**
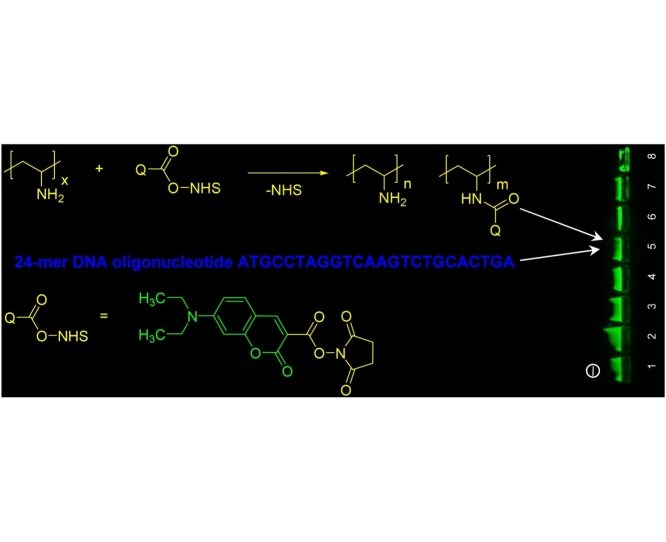

**Specifications Table**

**Table:** 

**Subject Area**	• *Biochemistry, Genetics and Molecular Biology*
**More specific subject area:**	*Gene delivery, gene therapy, genetic engineering*
**Method name:**	*Labeling of polymeric amines with coumarin tag*
**Name and reference of original method**	*T. Higashijima, T. Fuchigami, T. Imasaka, N. Ishibashi, Determination of amino acids by capillary zone electrophoresis based on semiconductor laser fluorescence detect ion, Anal. Chem. 64 (1992) 711-714.*
**Resource availability**	*NA*

## Method details

Study of the interaction between nucleic acids (DNA, RNA) and synthetic polymers is important in elaboration of new methods in genetic engineering and in gene therapy. Gel electrophoresis is a convenient method for detection of this interaction and for estimation of optimal nucleic acid – polymer ratio. Fluorescence dyes are usually applied to visualize the electrophoresis results. There are two main ways to introduce fluorescence agent in to electrophoretic system: to use intercalation agents like ethidium bromide [1] or to tag nucleic acid or synthetic polymer. The intercalation reaction is effective for double stranded nucleic acids and it is often complicated with competition from nucleic acid - polymer interaction which decreases emission [2]. Introduction of fluorescence fragment into oligonucleotides allows monitoring of their movement during electrophoresis and comparison between nucleic acid - polymer and blank system gives information about complexing. Fluorescence tagging of the synthetic polymer gives information from the opposite side, allows to see unbounded polymer and to use various non-tagged samples of nucleic acids.

Succinimidyl ester of 7-(diethylamino)coumarin-3-carboxylic acid (SECCA) was recommended as a tool for introducing fluorescent coumarin groups into aminoacids and proteins [3] but this reagent is not actively applied in labeling of synthetic polymeric constructions. We have used SECCA in synthesis of amine containing fluorescent dye for monitoring of siliceous structures in living organisms [4]. In this paper we describe procedures for tagging poly (vinyl amine) (PVA) and polyethylenimine (PEI) with SECCA and demonstrate gel electrophoresis data with oligonucleotides and tagged polymers.

### Synthesis of the tagged polymers

The initial succinimidyl ester of 7-diethylaminocoumarin-3-carboxilic acid (SECCA) was prepared according to [5]. Polyethylenimine (PEI) 50% aqueous solution (Cat.No.: 33141, Mw 30–40 kDa) was purchased from SERVA Electrophoresis GmbH. Polyvinyl amine (PVA) (Mw 68 kDa) was obtained by alkaline hydrolysis of poly(vinyl formamide) [6,7]. Modification of the polymers proceeds in aqueous medium:
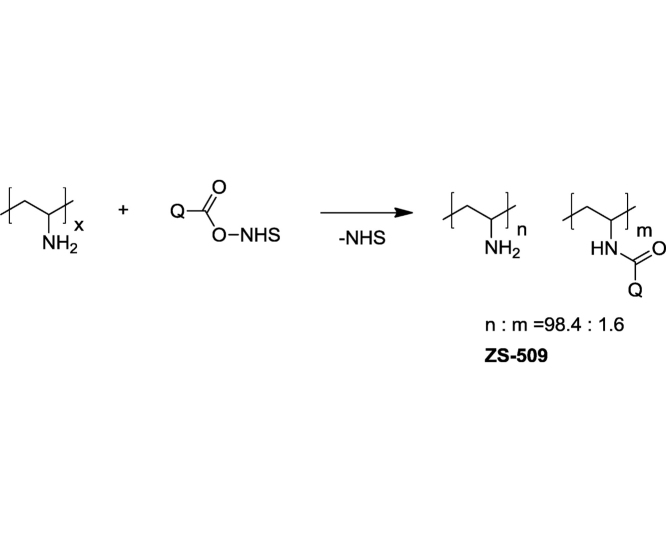

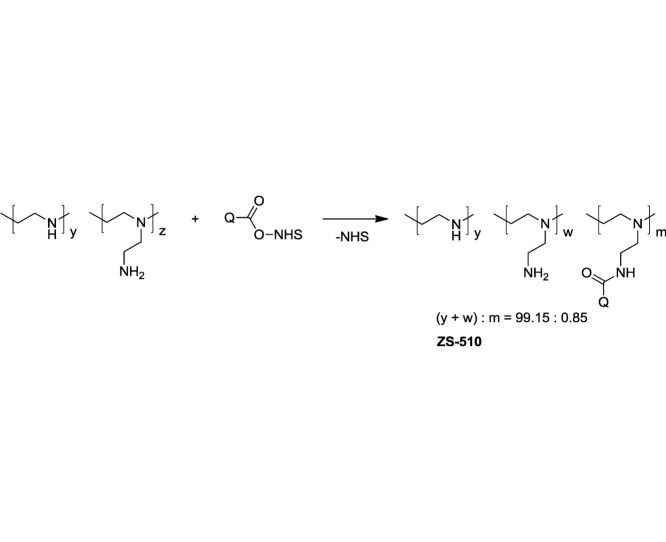

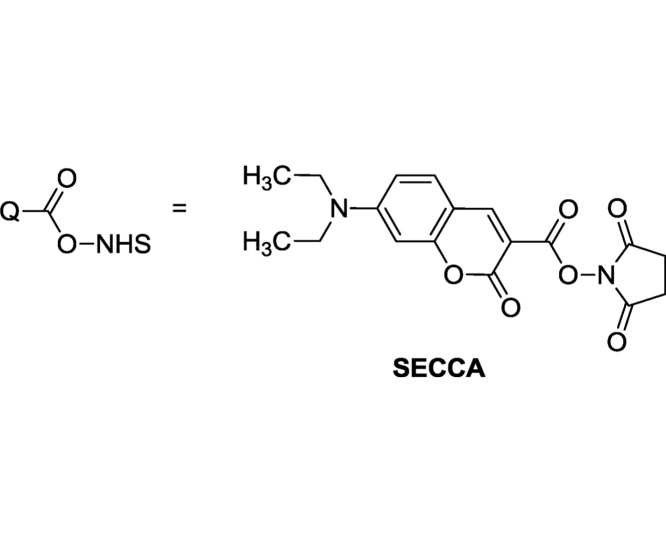


#### Fluorescent-tagged PVA

**Fig. 1:**
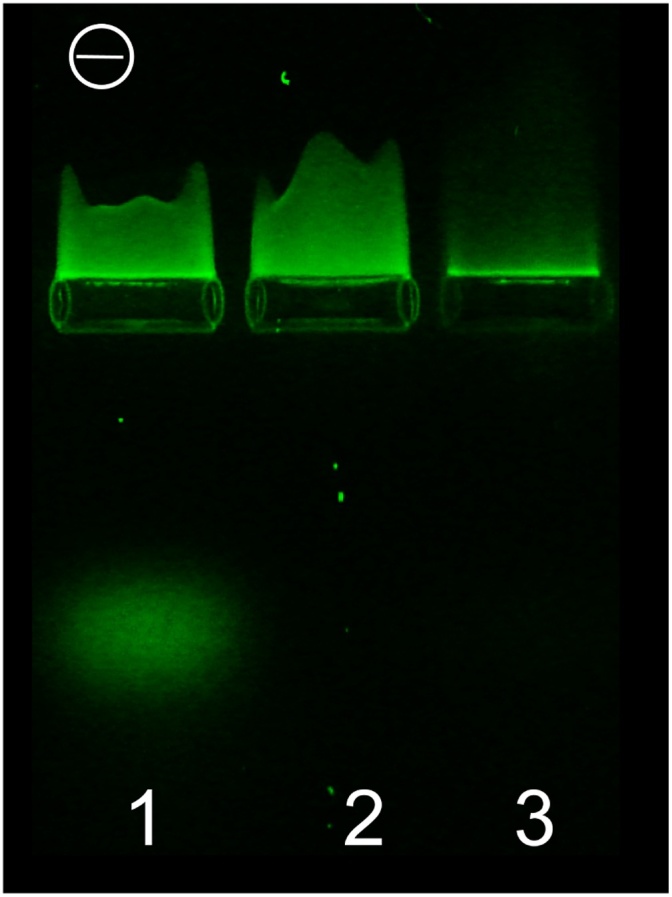
Gel electrophoresis data for ZS-509 sample with admixture of low-molecular dye (lane 1), pure ZS-509 and ZS-510 samples (lanes 2 and 3 respectively). Excitation – 395 nm.

To a solution of PVA (101.06 mg, 2.35 mmol) in 2 mL of deionized water glacial acetic acid (114.7 mg, 1.91 mmol) was added. pH of the solution was adjusted to 7.5 with gradual addition of 1.175 mL of 1 M NaOH. After that a solution of SECCA in DMF (96 mM, 0.727 mL, 0.0698 mmol) was added to the clear polymer solution. The resulted turbid mixture was shaken for 4 h at room temperature (23 °C) and left for 18 h in a dark place. The course of the reaction was checked with TLC on silica gel plates illuminated with a 395 nm flashlight (CH_3_OH:CH_2_Cl_2_: 25% aq. NH3 = 2:1:1). At the end of the process turbidity of the mixture became much lower. The solution was filtered through a 1 cm layer of alumina oxide. The solvent was evaporated under reduced pressure, the residue was mixed with 5 mL of distilled water and acidified to pH 1–2 with concentrated hydrochloric acid and dialyzed in Biotech CE Tubing (MWCO: 500–1000) against distilled water during seven days. The degree of purification of the polymer from the dye was checked by TLC illuminated with a 395 nm flashlight and with gel electrophoresis (Fig. 1). Then the polymer solution was filtered with 0.45 μm filter and freeze-dried.

#### Fluorescent-tagged PEI

PEI (128.5 mg, 2.98 mmol) was fluorescently tagged with SECCA analogously to PVA. The amounts of reagents were proportionally increased.

Fluorescent labeling of polymer was quantified according to the Beer-Bouguer-Lambert’ law and taking the molar extinction coefficients of 7-diethylaminocoumarin-3-carboxilic acid as standard [8] (34,000 cm^−1^ M^−1^ at 409 nm). UV–vis absorption measurements were carried out using a Cintra 20 UV/vis spectrophotometer (Sangji, Korea). The modified PVA contains 1.6 mol % of the fluorescent units and PEI – 0.85 mol %.

### Absorption and fluorescence spectra of the tagged polymers

**Fig. 2:**
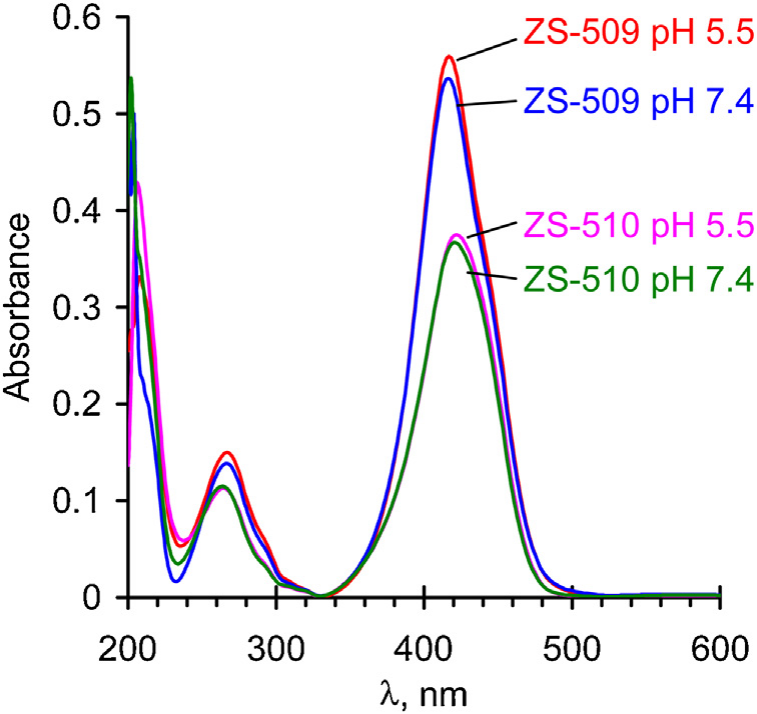
Absorption spectra of ZS-509 and ZS-510 at different pH. Polymer concentration is 50 μg/mL.

**Fig. 3:**
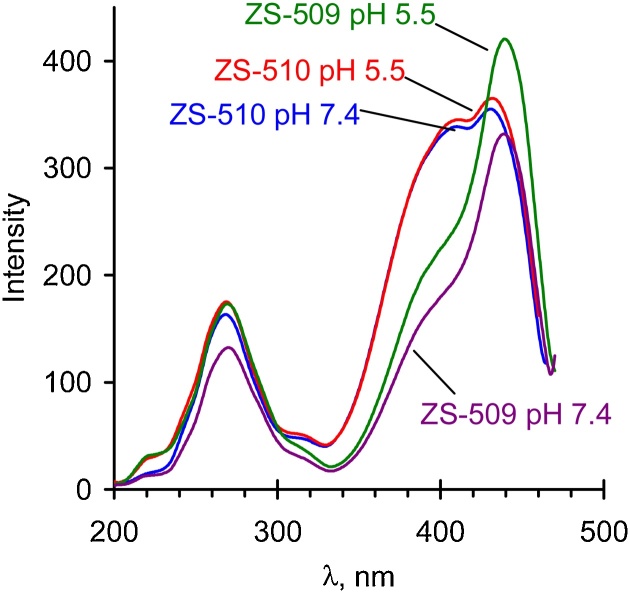
Excitation spectra of ZS-509 and ZS-510 at different pH at 483 nm emissions. Polymer concentration is 50 μg/mL.

**Fig. 4:**
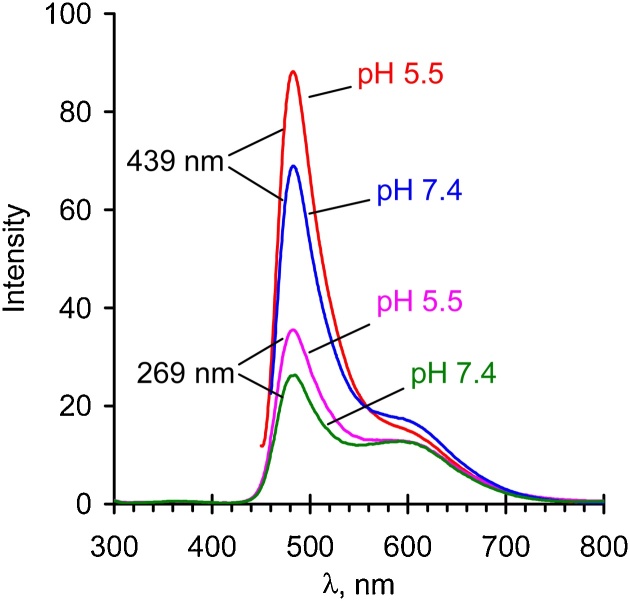
Fluorescence spectra for 50 μg/mL solutions of ZS-509 at different pH. Excitation at 269 and 439 nm.

**Fig. 5:**
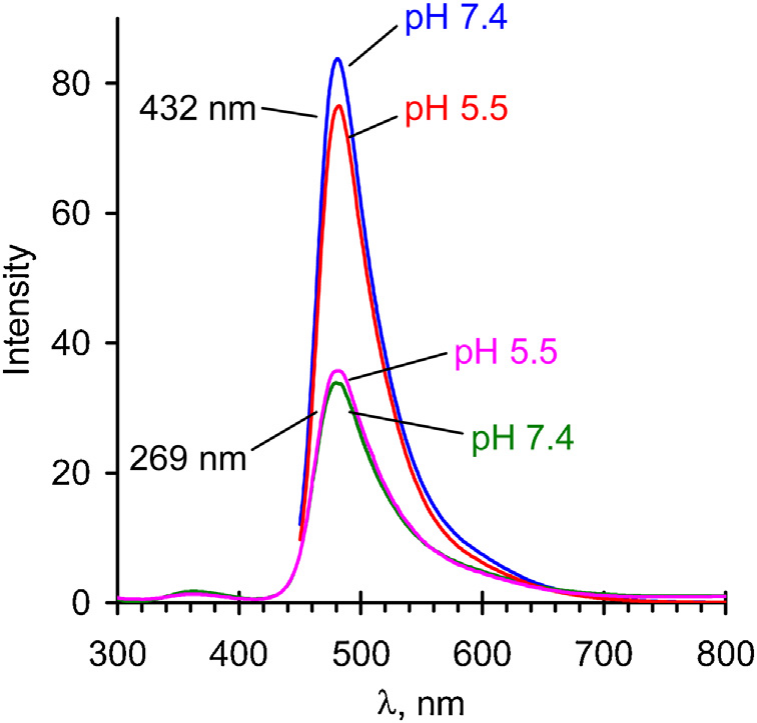
Fluorescence spectra for 50 μg/mL solution of ZS-510 at different pH. Excitation at 269 and 432 nm.

Photoluminescence spectra were recorded at 25 °C using Perkin-Elmer LS-55 instrument. All excitation spectra were recorded using the wavelength of the emission maximum as the emission wavelength. For absorption and fluorescence measurements the path length of the quartz cuvette was 1 cm. The experiments were carried out in 40 mM Tris acetate buffer and pH was adjusted to 5.5 and 7.4 with acetic acid. The obtained results (Fig. 2, Fig. 3, Fig. 4, Fig. 5) show two excitation peaks in UV (250–290 nm) and visible (370–450 nm) regions. The spectra shape and intensity are similar at pH 7.4 and 5.5 which allows to use the coumarin tag at physiological and weak-acid pH values.

### Study of polymer – nucleic acid interaction

24-mer DNA oligonucleotide ATGCCTAGGTCAAGTCTGCACTGA was purchased from Evrogen JSC (Russia). Electrophoresis experiments were performed with a Mini-Sub (7 × 10 cm) Cell GT System (Bio-Rad Laboratories, Inc.) with an ELF-4 power supply (DNA-Technology LLC) and TCP-20.LC transilluminator (Vilber Lourmat), operated at 254 nm. The gel running buffer was 40 mM Tris acetate (pH adjusted to pH 7.4) and 1 mM ethylenediaminetetraacetic acid (EDTA). A glycerol gel loading buffer was applied (0.5% sodium dodecyl sulfate, 0.1 M EDTA (pH = 7.4), 50% glycerol for 10x reagent).

**Fig. 6:**
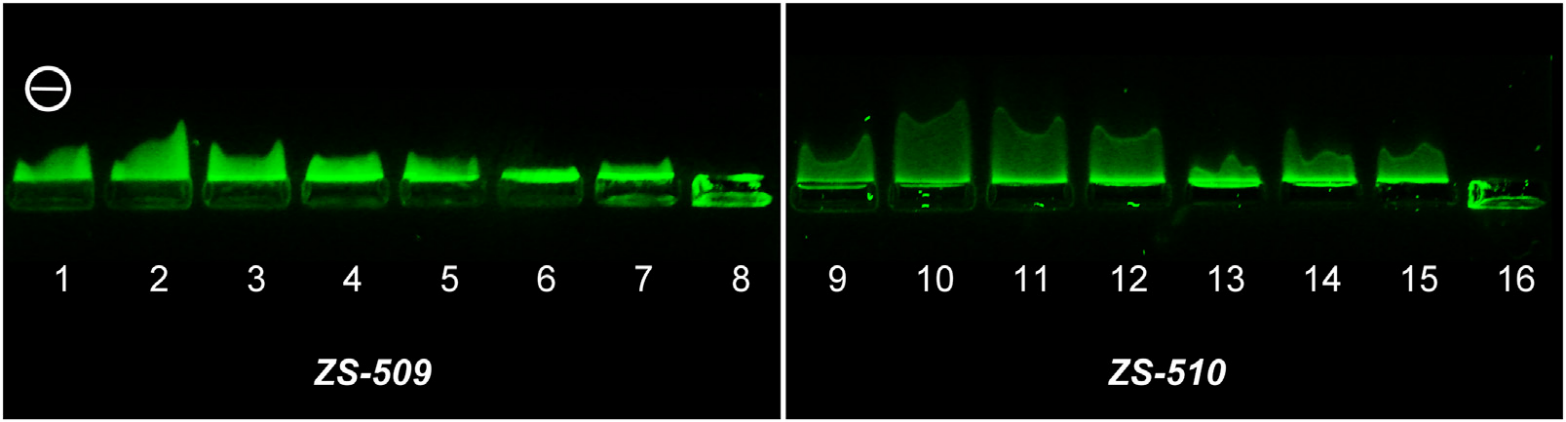
Gel electrophoresis data for ZS-509 and ZS-510 complexes with 24-mer DNA oligonucleotide. Concentration and volumes of the components are presented in Table 1. Excitation – 395 nm.

DNA and polymer solutions were mixed according to Table 1 and studied with gel electrophoresis after 30 min. The results (Fig. 6) show movement of the polymers to negative electrode (lanes 1, 2, 9, and 10) due to positive charge of the polymer. Decrease of N: P ratio (increase of DNA content in the mixture) results in decrease of the positive trail and at N:P = 14:1 the whole stained polymer remains in the starting pocket which corresponds to binding of the whole polymer with DNA giving a neutral complex.

**Table 1:** Conditions of gel electrophoresis for ZS-509 and ZS-510 complexes with 24-mer DNA oligonucleotides (concentration of the polymers – 2 mg/mL).

Lane #	Polymer	DNA concentration, μM	DNA volume, μL	Polymer volume, μL	N:P ratio
1	ZS-509	–	0	2	–
2		–	0	3	–
3		10	2	3	290:1
4		10	4	3	145:1
5		10	5	2.5	100:1
6		10	5	2	77:1
7		20	5	2	39:1
8		20	7	1	14:1

9	ZS-510	–	0	2	–
10		–	0	3	–
11		10	2	3	290:1
12		10	4	3	145:1
13		10	5	2.5	100:1
14		10	5	2	77:1
15		20	5	2	39:1
16		20	7	1	14:1

Thus, modification of polymeric amines with succinimidyl ester of 7-diethylaminocoumarin-3-carboxilic acid is a convenient method to study interaction between polymer and nucleic acids. Behavior of the tagged polymers during gel electrophoresis is easy visualized by fluorescence and this allows to optimize polymer – nucleic acid ratio in gene delivery and gene engineering experiments.
